# SubPc-Br/BiOI S-scheme heterojunctions: efficient charge separation for enhanced photocatalytic degradation of tetracycline†

**DOI:** 10.1039/d5ra02536b

**Published:** 2025-06-02

**Authors:** Yijian Zhou, Mengting Ji, Shengqian Liang, Jiahang Song, Haotian Wu, Enzhou Liu, Bing Wang, Chen Wang, Bo Zhou, Zhuo Li

**Affiliations:** a School of Chemical Engineering, Northwest University Xi'an 710069 China wangbingphd@163.com lz@nwu.edu.cn; b International Scientific and Technological Cooperation Base for Clean Utilization of Hydrocarbon Resources, Shaanxi Key Laboratory for Carbon Neutral Technology, Chemical Engineering Research Center of the Ministry of Education for Advance Use Technology of Shanbei Energy, Shaanxi Research Center of Engineering Technology for Clean Coal Conversion, Collaborative Innovation Center for Development of Energy and Chemical Industry in Northern Shaanxi Xi'an 710069 China; c Technological Institute of Materials & Energy Science (TIMES), Xijing University Xi'an 710123 China; d Institute of Modern Physics, Shaanxi Key Laboratory for Theoretical Physics Frontiers, Northwest University Xi'an 710069 China

## Abstract

Constructing S-scheme heterojunctions is an effective strategy to enhance charge separation efficiency. In this study, for the first time, boron subphthalocyanine bromide (SubPc-Br) was formed by self-assembly on the surface of layer BiOI to form an S-scheme heterojunction (SubPc-Br/BiOI) through halogen bonding and π–π stacking interactions. Experimental results demonstrate that the SubPc-Br/BiOI composite enhances tetracycline removal efficiency by a factor of 1.6 compared to pure BiOI. Notably, after five cycles, the composite still maintains a high tetracycline removal rate, which is 2.62 times that of pure BiOI. DFT and TDDFT theoretical calculations, combined with synchrotron X-ray photoelectron spectroscopy (XPS) under simultaneous illumination, indicate that the internal electric field generated between the [Bi_2_O_2_] layer and the SubPc-Br macrocycle plays a dominant role in charge separation, while interfacial electron transfer contributes to the constitution of the S-scheme heterojunction. Moreover, the combination of molecular dynamics simulations (MD), Fukui function calculations, and HPLC-MS detection reveals the mechanism of pollutant degradation. This study introduces an innovative strategy for the construction of BiOI-based S-scheme heterojunctions.

## Abstract 2

Constructing S-scheme heterojunctions is an effective strategy to enhance charge separation efficiency.

## Graphical abstract

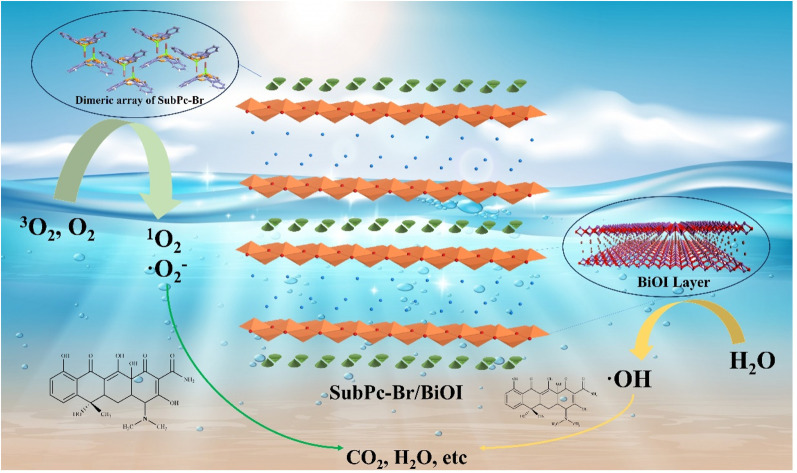

## 1. Introduction

Advanced oxidation processes represent an innovative and efficient technology in contemporary wastewater treatment.^1–3^ This strategy is intended to completely degrade recalcitrant organic pollutants in wastewater through an array of complex chemical and physical processes, thereby opening up a new avenue for environmental protection. The essence of this process involves the synergistic use of photoelectric irradiation, catalysts, and oxidizers to generate highly oxidizing active reactive oxygen species (ROS), including hydroxyl radicals (·OH) and superoxide radicals (·O_2_^−^), photogenerated holes, and other reactive species.^4,5^ These reactive species rapidly interact with organic pollutants, breaking them down into less or non-toxic small molecules and, in some cases, completely mineralizing them into carbon dioxide and water.^6,7^ Common advanced oxidation processes encompass ozone oxidation,^8^ Fenton oxidation,^9^ photocatalytic oxidation,^10^*etc.* Among these methods, ozone oxidation is characterized by relatively high production costs and limited solubility of ozone in water, leading to reduced efficiency.^11^ Furthermore, Fenton oxidation typically requires acidic conditions, which not only increases treatment costs and complexity but also makes the iron ions in Fenton reagents prone to secondary contamination.^12,13^ In contrast, the photocatalytic oxidation process operates under mild conditions, demonstrating superior environmental friendliness and offering broader application prospects.^14^ This is reflected in its low equipment requirements, low energy consumption, absence of secondary pollution, ability to utilize solar energy for cost reduction, promising development potential, and effectiveness in degrading refractory pollutants.^15^ Since Fujishima *et al.* first achieved photocatalytic water splitting using a TiO_2_ semiconductor single-crystal electrode in 1972, semiconductor photocatalyst research has remained at the forefront of global scientific innovation.^16^ Various semiconductor, such as TiO_2_,^17^ ZnO,^18^ CdS,^19^ and ZnS,^20^ have been continuously developed and reported. Nevertheless, the restricted light absorption spectrum and the swift recombination of photogenerated charge carriers frequently impede the pragmatic application of these photocatalysts.^21^

Halogen bismuthates (X = Cl, Br, I) have attracted considerable interest in the photocatalytic field, attributed to its broad light absorption range and optimal bandgap width.^22,23^ The primary factor lies in the effective promotion of photogenerated charge separation by the internal electric field distribution within the BiOX photocatalyst.^24^ Furthermore, the concurrent increase in halogen atoms, the bandgap structure of the BiOX photocatalyst narrows. Consequently, in theory, BiOI possesses the smallest bandgap (1.75 eV) among the BiOX photocatalysts, thereby exhibiting an excellent visible light response and catalytic activity.^25,26^ However, the [Bi_2_O_2_]^2+^ layer in BiOI is physically constrained by the strong coulombic interaction between electron–hole pairs, which can lead to excitonic effects. The generation of excitons competes with the generation of charge carriers, which significantly impacts the photocatalytic efficiency.^27^ To address these challenges, researchers often resort to modifying BiOI. A variety of modification techniques are commonly adopted, including the deposition of precious metals, doping with elements, forming composite semiconductors, enhancing surface photosensitivity, and regulating morphology.^28,29^ Among these, the establishment of heterogeneous structures holds significant advantages. Specifically, the construction of S-scheme heterojunctions is currently the most effective method.^30^ For instance, Wang *et al.*^31^ design and synthesis of a 2D/2D van der Waals Bi_2_MoO_6_/BiOI heterogeneous junction for CO_2_ reduction. This structure facilitates charge transfer and enables effective charge separation and powerful redox capabilities through a large-area interface van der Waals heterojunction. Additionally, the introduction of Bi_2_MoO_6_ further reduces the threshold for CO_2_ photoreduction, thus improving photocatalytic activity. Wu *et al.*,^32^ for the first time, designed and constructed a new type of ultrathin hollow nanotube Bi_2_Sn_2_O_7_/Bi_4_O_5_I_2_ discrete structure modified by quantum dots. The design and synthesis of the S-scheme heterojunction enhances the movement of photoexcited carriers and the spatial separation of redox reaction sites. An *et al.*^33^ designed a vacancy-amplified BiOI/g-C_3_N_4_ S-scheme heterojunction to improve the activation efficiency of O_2_ for bisphenol A (BPA) degradation in water treatment. The results demonstrate that the introduction of vacancies can modulate the electronic structure and enhance the endogenous electrostatic field in the S-scheme heterojunction. Consequently, this broadens the range of light absorption and further enhances the efficiency of photogenerated charge separation. Lou *et al.*^34^ regulated the electronic band structure of Bi_2_O_2_CO_3_ through Co doping and promoted the growth of BiOI on its surface *via* shared Bi atoms, thereby developing a Co-Bi_2_O_2_CO_3_/BiOI S-scheme heterojunction. This structure features an atomically close-contact interface, which markedly enhances optical absorption, carrier separation, and transmission efficiency. However, to date, there have been no reports of the spontaneous assembly of S-scheme heterojunctions on the exterior of BiOI using organic supramolecular photosensitizers.

Compared with inorganic semiconductor sensitizers, organic semiconductor photosensitizers can broaden the solar absorption range of photocatalysts and enhance their utilization efficiency. Their molecular structures can be designed as needed to optimize optical and electrical properties, thereby significantly improving the generation efficiency of photoinduced charge carriers. Additionally, they are capable of enhancing the surface properties of photocatalysts, thereby enhancing the adsorption capacity and reaction efficiency of substrates.^35^ Commonly employed photosensitizers encompass a spectrum of materials, including phthalocyanines (Pcs), porphyrins, and sub phthalocyanines (SubPcs), *etc.*^35–37^ Metal phthalocyanine complexes are characterized by a unique metal-N4-chelate (MN_4_) 18-π electron conjugated structure, which imparts them with stable chemical properties. These complexes exhibit both electron-donating and electron-accepting capabilities, and are frequently utilized as non-radical oxidants in photocatalytic systems. ^35,38,39^ Compared to porphyrins, the conjugated structure of phthalocyanines (Pcs) can enhance biomimetic catalytic oxidation performance through modifications with various substituents.^40^ Sub phthalocyanines (SubPcs) represent the simplest homologues of phthalocyanines, possessing a conical structure absent in phthalocyanines. This distinctive conical shape, primarily attributed to the planarity deviation induced by tetrahedral coordination, confers upon SubPcs the capability to exhibit nonlinear optical properties through the second and third-order harmonics of phthalocyanines.^41,42^ Additionally, SubPcs possess a 14-π electron conjugated structure, distinct from phthalocyanines, which endows them with excellent photoreactivity and stability. Furthermore, subphthalocyanine exhibits enhanced stability in water and a reduced tendency to aggregate compared to phthalocyanine.^43,44^ In the realm of photocatalytic oxidation, SubPcs compounds provide a novel alternative for constructing heterojunctions to augment photocatalytic capability.

In this research, the BiOI photocatalyst was manufactured using a direct hydrothermal method. Subsequently, the SubPc-Br/BiOI photocatalyst, which exhibits improved efficiency and stability, was prepared through a rotary evaporation technique. The composite photocatalyst was characterized using various techniques. Through molecular dynamics simulations, the dynamic behavior of SubPc-Br molecules and antibiotic molecules on the BiOI surface was elucidated. Meanwhile, the results of IGM analysis reveal a weak interaction between SubPc-Br and BiOI, thereby indicating that the two components are not merely undergoing simple physical mixing. The calculations of the ground state 3D difference charge, in conjunction with experimental outcomes, indicate the presence of an internal electrostatic field across the SubPc-Br and BiOI semiconductors. This internal electric field stimulates the separation of photogenerated charges and mitigates the exciton effect in BiOI, thereby endowing the SubPc-Br/BiOI system with exceptional photocatalytic effectiveness. Moreover, the potential biodegrade pathways of tetracycline (TC) were investigated using Liquid Chromatography-Mass Spectrometry (LC/MS) and Fukui function analysis. The high-performance degradation of TC by the SubPc-Br/BiOI photocatalyst highlights the potential application of S-scheme heterojunctions in water environmental purification.

## 2. Experimental section

### 2.1. Materials

The materials utilized in this study were analytical grade and were obtained from Aladdin.^45^ No further purification was deemed necessary prior to their use.

### 2.2. Preparation of photocatalytic materials

#### 2.2.1. Preparation of SubPc-Br

The synthesis was conducted following the previously reported method.^46^

#### 2.2.2. Preparation of BiOI

Bi(NO_3_)_3_·5H_2_O was dissolved within 20 mL of ethylene glycol under churning the mixture until transparency is observed. Subsequently, 60 mL of a KI aqueous solution, with the same stoichiometric amount of Bi(NO_3_)_3_·5H_2_O, was incrementally added dropwise to the homogeneous mixture. Subsequently, the mixture was stirred at ambient temperature for 30 minutes to achieve uniformity. Subsequently, the solution was transferred to a 100 mL autoclave and heated at 150 °C in an oven for 12 hours. The resulting precipitate was cleansed repeatedly with water and EtOH, followed by drying in a constant temperature drying chamber at 80 °C for 3 hours. This process yielded the final product as a brick-red powder of BiOI.

#### 2.2.3. Preparation of SubPc-Br/BiOI

A solution containing 50 mg SubPc-Br in 100 mL toluene was subjected to ultrasonication for 30 minutes. BiOI was subsequently introduced into the solution at mass ratios of 1 : 10, 1 : 25, 1 : 50, and 1 : 75. The resultant mixture was then subjected to rotary evaporation at 80 °C for 4 hours until complete toluene evaporation. The obtained SubPc-Br/BiOI composites were scoured with ultra-pure water and vacuum-dried.

### 2.3. Characterization tests

For further details on the characterization tests, please refer to ESI S1.1.†

### 2.4. Photocatalytic activity evaluation and cyclic performance test

The experiment utilized wastewater with a mass concentration of 20 mg L^−1^. The mixture was stirred at 25 °C under atmospheric pressure and illuminated with a xenon lamp (watt 300 W). Samples were taken every 10 minutes, and the absorbance of the samples was precisely surveyed using a UV-2550 to assess the activity of the photocatalyst. The degradation efficiency was calculated using the formula:^47^1
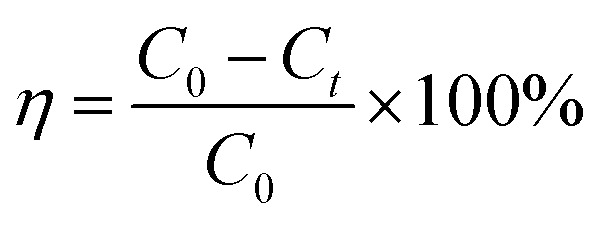


In this context, *η* denotes the degradation efficiency, *C*_0_ represents the original concentration, and *C*_t_ corresponds to the residual concentration.

Catalytic cycle stability is also a crucial indicator for evaluating catalyst performance. To assess this, the following procedure was conducted. Initially, the solution was filtered post the initial photodegradation of antibiotics to harvest the resulting photocatalyst. This photocatalyst was then rinsed with deionized water followed by anhydrous ethanol, followed by centrifugal separation to isolate the remaining catalyst, which was subsequently dried for 3 hours at 80 °C in a vacuum drying chamber. The dried photocatalyst was then retrieved and utilized for the subsequent photocatalytic experiments, with all conditions maintained consistent with the initial setup. This process was iterated during each cycle until the completion of the consecutive cycles.

### 2.5. Computational methods

The reciprocity between SubPc-Br molecules dissolved in water and the surface of BiOI (001) was investigated using molecular dynamics simulations within supercell models with (6 × 6) periodic boundary conditions.^19,48^ The condensed phase was modeled using the UNIVERSAL force field.^49^ Prior to molecular dynamics (MD) simulations, the system was geometrically optimized to ensure accurate results.^50,51^ Molecular dynamics simulations of the supramolecular self-assembly system were performed using the Forcite program with the UNIVERSAL force field and NVT ensemble. The simulation was conducted for a total time of 1000 ps, with a time step of 0.1 fs.^52^ This analysis encompassed the communication between SubPc-Br on the BiOI surface, the local action of antimicrobial agents (tetracycline, TC) on the SubPc-Br and BiOI surfaces.

The ground-state properties were evaluated using density functional theory (DFT) with the B3LYP functional and 6-31G basis set. The Castep module was employed to evaluate the band structure, density of states, work function, Fermi energy level, 3D differential charge density, and carrier mobility for BiOI, SubPc-Br, and the SubPc-Br/BiOI complex.^53^ Subsequently, the Fukui function was calculated on the optimized TC.fch files using Multiwfn software.^54,55^ Based on the obtained mass spectrometry data and Fukui function analysis, the potential degradation pathways of antibiotics were deduced. Further details regarding the theoretical calculations are listed in the ESI S1.2.†^56^

## 3. Results and discussions

### 3.1. Morphological structure characterization

**Fig. 1:**
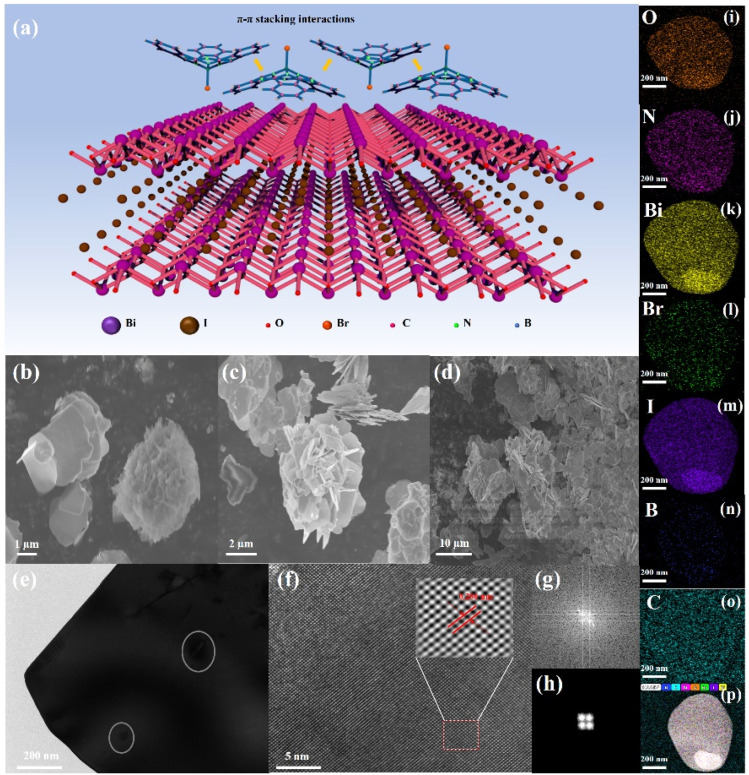
(a) Diagram illustration of the construction of the SubPc-Br/BiOI heterogeneous junction catalyst. Scanning electron microscopy (SEM) images showing pristine, (b and c) BiOI and (d) SubPc-Br/BiOI (1 : 25) composite. Transmission electron microscopy (TEM) images showing (e) SubPc-Br/BiOI (1 : 25) composite, (f) lattice fringes of the BiOI (110), (g and h) he FFT and inverse FFT images showing BiOI. (i–p) TEM-EDS elemental mapping images showing SubPc-Br/BiOI (1 : 25) composite.

Fig. 1a illustrates the self-assembly of subphthalocyanine into a supramolecular array on the BiOI surface through halogen bonding and π–π stacking interactions, forming an organic–inorganic supramolecular photosensitive system. Previous studies have demonstrated that constructing such supramolecular systems and expanding the π-conjugation can significantly extend the photoactivation lifetime of photosensitizers, increase carrier mobility, and enhance the carrier transfer efficiency between photosensitizers and catalysts.^17,51^ The microstructure of the resulting products was characterized using SEM and TEM. As shown in Fig. 1b–d, pure BiOI exhibited a 2D lamellar structure with a size of approximately 8 μm, with some degree of restacking observed (Fig. 1b). The morphology of SubPc-Br/BiOI was very similar to that of BiOI, indicating that the rotational evaporation method used for SubPc-Br doping did not significantly change the morphology of BiOI (Fig. 1d). The degree of restacking in BiOI was notably reduced upon adding SubPc-Br (Fig. 1d). Further characterization of BiOI and SubPc-Br/BiOI samples was performed using TEM. Fig. 1e–g display TEM images showing SubPc-Br/BiOI at a ratio of 1 : 25, with Fig. 1e showing SubPc-Br nanocrystals (indicated by a green circle) dispersed on a BiOI sheet. The lattice fringes of the BiOI (110) plane are visible in Fig. 1f, with a spacing between lattice points of 0.285 nm, consistent with previous findings.^57^Fig. 1g and h displays the FFT and inverse FFT images showing BiOI. The findings indicate that the synthesized BiOI retains its original tetragonal phase, signifying that the lattice structure of the pristine material remains unaltered following the incorporation of SubPc-Br. Fig. 1i–p presents the elemental mapping results, which reveal the uniform distribution of O, N, Bi, Br, I, B, C and all elements inner the SubPc-Br/BiOI composite materials. This observation confirms the achieved preparation in the SubPc-Br/BiOI composite materials.

**Fig. 2:**
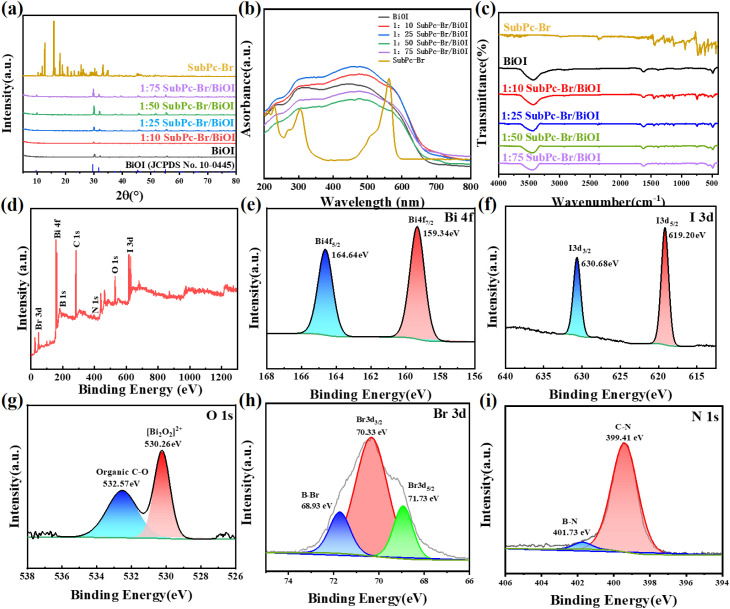
(a) X-ray diffraction (XRD) patterns of SubPc-Br and the synthesized materials at various mass ratios, (b) ultraviolet-visible (UV-Vis) absorption spectra of SubPc-Br and materials at different mass ratios, (c) Fourier-transform infrared (FT-IR) spectral data of the prepared samples at different mass ratios, (d) survey X-ray photoelectron spectroscopy (XPS) spectral characteristics of SubPc-Br/BiOI (1 : 25) composite, (e–h) XPS spectral characteristics of Bi 4f, I 3d, O 1s, Br 3d for SubPc-Br/BiOI (1 : 25) composite, (i) XPS spectra of N 1s for SubPc-Br/BiOI (1 : 25) composite.

The samples' crystal structure was determined by XRD. As elucidated in Fig. 2a, the peaks align with the standard card for BiOI [JCPDS file no. 10-0445], in agreement with the literature.^31^ The peaks located at 9.68°, 29.79°, 31.78°, 45.51°, 51.43°, and 55.34° correspond to the (001), (012), (110), (200), (114), and (212) crystal planes, respectively, with the (012) plane exhibiting the most intense diffraction peak, indicating preferential growth in this direction.^58^ Upon the incorporation of SubPc-Br onto the BiOI photocatalyst, no extrinsic peaks corresponding to SubPc-Br were detected in the XRD spectrum. This absence is probably a result of the low loading amount and excellent dispersion by SubPc-Br on the BiOI photocatalyst surface. The BiOI photocatalyst within the SubPc-Br/BiOI composite retains its crystalline nature.^56^

The samples exhibited optical properties examined *via* UV-vis spectroscopy. As shown in Fig. 2b, the edge of absorption for BiOI is approximately 650 nm. In accordance with the Gouterman orbital model, the minor absorption peak at 281 nm is associated with the Soret band, reflecting the S0 → S2 B transitions. The strong absorption peak at 483 nm, which is approximately double intense as the B transition, represents the S0 → S1 Q band transition.^53,54^ Compared to BiOI, the SubPc-Br/BiOI composite exhibited an extended light absorption range. Notably, as shown in Fig. 2b, the visible light absorption edge of SubPc-Br/BiOI was significantly red-shifted relative to BiOI. This red shift is likely attributed to the introduction of SubPc-Br, which altered the electron cloud density of BiOI through changes in intermolecular van der Waals interactions, thereby shifting the absorption peak to longer wavelengths. This observation also confirms the successful assembly of SubPc-Br onto the BiOI surface.

Fourier-transform infrared spectroscopy (FT-IR) was used to analyze the chemical structure of the synthesized materials, and the results are shown in Fig. 2c. In the spectrum of pure BiOI, the peak at 490 cm^−1^ is attributed to the stretching vibration mode of the Bi–O bond. The peak at 3400 cm^−1^ corresponds to the typical stretching frequency absorption of the –OH group.^59^ After depositing SubPc-Br onto the BiOI surface *via* a rotary evaporation technique, the SubPc-Br/BiOI photocatalysts exhibited characteristic absorption peaks in the infrared spectrum that can be attributed to SubPc-Br. Overall, the peak at 741 cm^−1^ is likely due to the stretching vibration of aromatic rings, while the peak at 679 cm^−1^ is associated with the bending vibration of aromatic rings. The frequency at 1447 cm^−1^ is related to the stretching vibration of the carbonyl group (C=O). The series of peaks in the range of 940–1500 cm^−1^ correspond to the stretching and bending vibrations of C–C, C–N, and C–H bonds in the macrocyclic framework. Additionally, a weak peak at 616 cm^−1^ was observed, which is attributed to the stretching vibration of the B–Br bond in SubPc-Br.^60^ These changes further indicate that SubPc-Br was successfully loaded onto the BiOI surface without altering the structure and chemical integrity of BiOI.

**Fig. 3:**
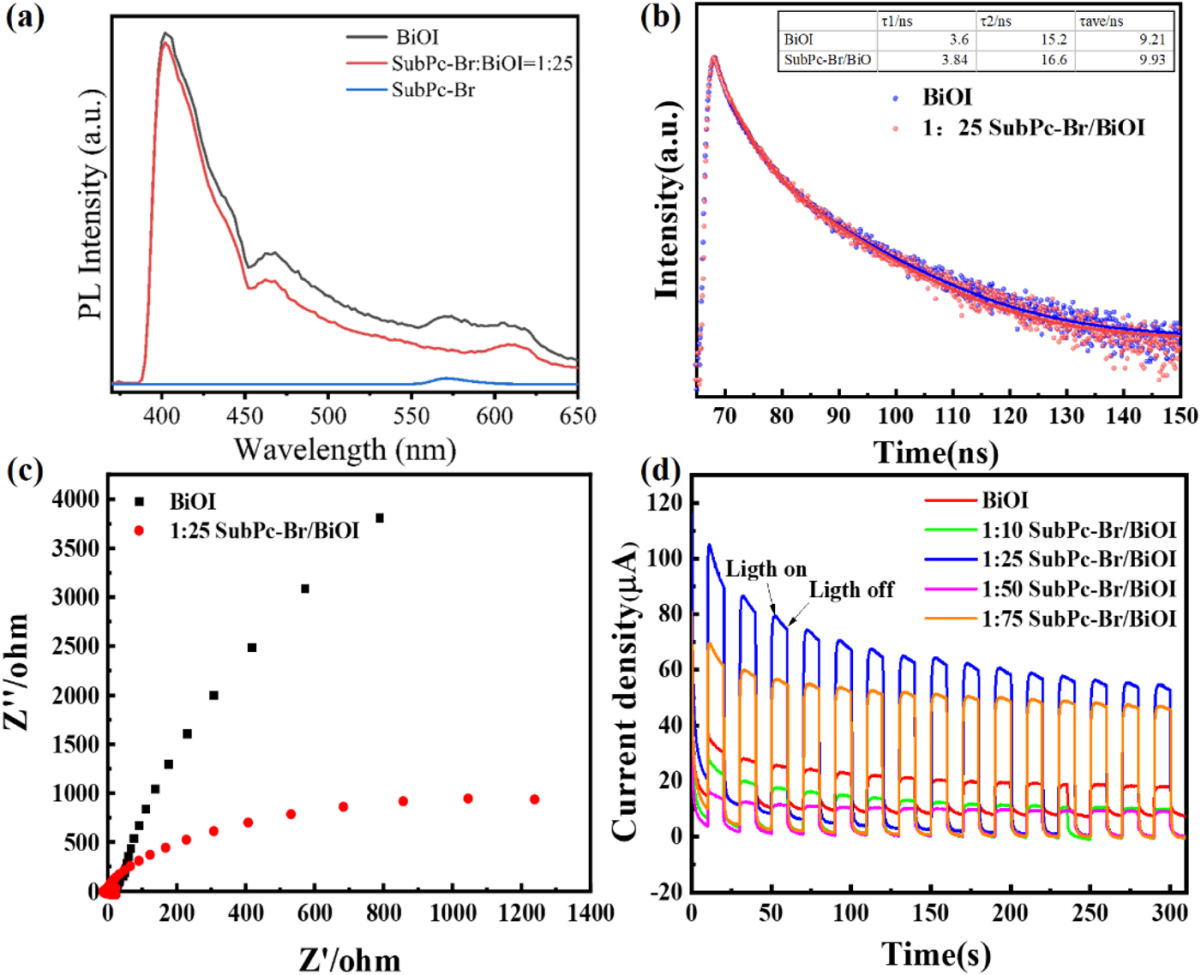
(a) PL spectroscopy of BiOI and SubPc-Br/BiOI (1 : 25), (b) TRPL decay spectrum, (c) EIS Nyquist curve, and (d) BiOI and SubPc-Br/BiOI (1 : 10, 1 : 25, 1 : 50, 1 : 75).

XPS was applied to characterize the elemental makeup and oxidation valence states of the elements in the SubPc-Br/BiOI composite. The XPS signals confirmed the identification of the elemental constituents of the composite include Bi, O, I, C, N, Br, and B (Fig. 2d). Specifically, the Bi 4f spectrum displayed two peaks at 164.64 eV and 159.34 eV (Fig. 3e), which were caused by the Bi 4f_5/2_ and Bi 4f_7/2_ spin–orbitals, respectively. This finding indicates the presence of Bi^3+^ in BiOI.^61^ In Fig. 2f, the I 3d spectrum of the SubPc-Br/BiOI composite exhibits peaks at 630.68 eV and 619.20 eV, which are associated with the I 3d_3/2_ and I 3d_5/2_ states in the respective cases. Regarding the O 1s spectrum shown in Fig. 2g, it can be deconvoluted into two distinct peaks. The peak at 530.26 eV is ascribed to the Bi–O bond within the [Bi_2_O_2_] interlayer of BiOI. In contrast, the peak at 532.57 eV is primarily ascribed to the presence of adsorbed ·OH groups on the surface layer of the composite.^24^Fig. 2h illustrates that the peaks at 71.73 eV and 70.33 eV correspond to the Br 3d_5/2_ and Br 3d_3/2_ states. The peak at 68.93 eV indicates the presence of B–Br bonds in the system.^62^Fig. 2i illustrates the N 1s spectrum of the SubPc-Br/BiOI catalyst, revealing peaks at 399.41 eV and 401.73 eV attributed to C–N and B–N bonds. These XPS results further substantiate the uniform dispersion of SubPc-Br on the BiOI nanosheets.

Fig. 3a presents the PL emission spectra of BiOI, SubPc-Br : BiOI (1 : 25), and SubPc-Br. PL spectra reflect the efficiency of photogenerated carrier transfer and separation within the catalysts. Notably, SubPc-Br/BiOI (1 : 25) shows a slightly lower fluorescence peak intensity at 400 nm than pristine BiOI, along with a broader and redshifted emission peak. This implies enhanced separation and transfer efficiency of photoexcited charge pairs in the composite, possibly reducing photogenerated carrier recombination and indicating improved photocatalytic activity. In contrast, SubPc-Br alone exhibits relatively weak PL intensity across the entire wavelength range, highlighting its distinct optical characteristics compared to BiOI. These results suggest that SubPc-Br introduction may induce energy transfer or other interactions affecting BiOI's photoluminescence properties, and the SubPc-Br/BiOI (1 : 25) composite enhances catalytic performance by improving photogenerated carrier separation. Fig. 3b illustrates the TRPL decay spectra of BiOI and SubPc-Br/BiOI (1 : 25). The data reveal that SubPc-Br/BiOI (1 : 25) exhibits a longer decay time compared to pristine BiOI. The average decay times (*τ*_ave_) were calculated to further substantiate these findings. The decay parameters for BiOI were *τ*_1_ = 3.60 ns and *τ*_2_ = 15.21 ns, with an average longevit (*τ*_ave_) of 9.21 ns. In contrast, for SubPc-Br/BiOI (1 : 25), *τ*_1_ increased to 3.84 ns, *τ*_2_ to 16.60 ns, and *τ*_ave_ to 9.93 ns. These results confirm that SubPc-Br/BiOI (1 : 25) effectively suppresses the recombination of photogenerated carriers during photocatalytic reactions, thereby extending the contact time between the catalyst and pollutant molecules and enhancing the photocatalytic activity.^63^

The electron transfer resistance for BiOI and the SubPc-Br/BiOI (1 : 25) composite was analyzed using EIS, with the results presented in Fig. 3c. In the Nyquist plot, a larger semicircle diameter indicates a higher electron transfer resistance. The plot reveals that the semicircle diameter for SubPc-Br/BiOI (1 : 25) is significantly smaller than that for pristine BiOI, indicating that SubPc-Br/BiOI (1 : 25) composite exhibits significantly lower internal resistance compared to pure BiOI.^64^ This enhancement leads to an increased rate of charge separation and improved transmission efficiency of photogenerated electrons.

Fig. 3d presents the photocurrent response profiles of BiOI and SubPc-Br/BiOI. The photocurrent response strength of the catalysts is assessed by comparing the transient photocurrent density changes under identical conditions. It is evident that under illumination, the abrupt increase in photocurrent density for SubPc-Br/BiOI (1 : 25) signifies a superior capability to facilitate charge transfer and spatial separation. The recombination rate of photogenerated charge carriers is significantly curtailed.^61^ This indicates that SubPc-Br/BiOI holds broad application potential as an efficient photocatalyst.

### 3.2. Photocatalytic performance analysis

**Fig. 4:**
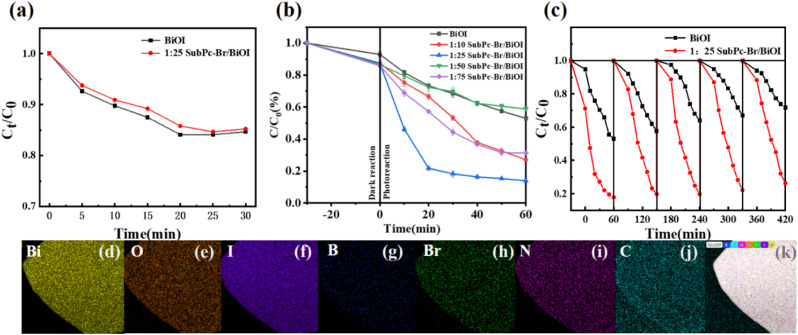
(a) BiOI and SubPc-Br/BiOI (1 : 25) TC adsorption experiment in dark conditions; (b) photocatalytic degradation of TC (10 mg L^−1^) using a 300 W xenon lamp with a 400 nm filter at 298 K; (c) SubPc-Br/BiOI (1 : 25) cycle experiment; (d–k) TEM-EDS element map of SubPc-Br/BiOI after loop (1 : 25).

A 30 minute dark reaction followed by a 60 minute light reaction. The differences between experiments are represented by error bars in the diagram. The standard deviation of less than 0.025 for all samples indicates the repeatability of the experiments (Table S1, ESI†). Initially, the adsorption–desorption behavior of SubPc-Br/BiOI (1 : 25) towards tetracycline (TC) was examined under dark conditions. Fig. 4a demonstrates that the concentration of tetracycline remains largely unchanged after a 30 minute light-independent reaction, revealing that the system has reached equilibrium in adsorption–desorption process. The photocatalytic efficiency of BiOI for tetracycline (TC) degradation was found to be only 47.1% (Fig. 4b). However, the addition of SubPc-Br significantly enhanced the photocatalytic decay of TC. Notably, SubPc-Br/BiOI (1 : 25) achieved a TC degradation rate of 84.4% upon illumination, demonstrating its superior efficiency over BiOI alone. The experimental results of BiOI and SubPc-Br/BiOI (1 : 25) were modeled using kinetics (l − ln(*C*/*C*_0_) = *kt*),^65^ where *k* is the pseudo-first-order rate constant (Fig. S2, ESI†). It was found that the apparent rate constant for SubPc-Br/BiOI (1 : 25) was 4.26 times enhanced compared to that of BiOI, demonstrating that photodegradation was the predominant factor contributing to the reduction of TC concentration throughout the reaction process. The results demonstrate that the introduction of SubPc-Br upgraded the photocatalytic traits of the sample.

Subsequently, the cyclic stability of the catalysts was assessed under identical experimental conditions through repeated photocatalytic experiments. As illustrated in Fig. 4c, after five cycles, the TC degradation rate was maintained at SubPc-Br/BiOI was sustained at 73.7%, whereas with BiOI alone, the TC removal rate dropped to 28.1%. This indicates that SubPc-Br/BiOI possesses enhanced stability in photocatalytic activity and has a longer lifespan in TC degradation *versus* BiOI. More insights into BiOI – based heterojunction – mediated TC degradation are provided in the Table S2, ESI.†

The stability of the SubPc-Br/BiOI photocatalyst was confirmed by SEM and EDS analysis. The elemental mapping from SEM-EDS (Fig. 4d–k) revealed the existence of Bi, O, I, B, Br, N, and C elements on the exterior surface of SubPc-Br/BiOI, which were distributed uniformly. These results demonstrate the relative stability of SubPc-Br/BiOI.

### 3.3. Theoretical calculation

**Fig. 5:**
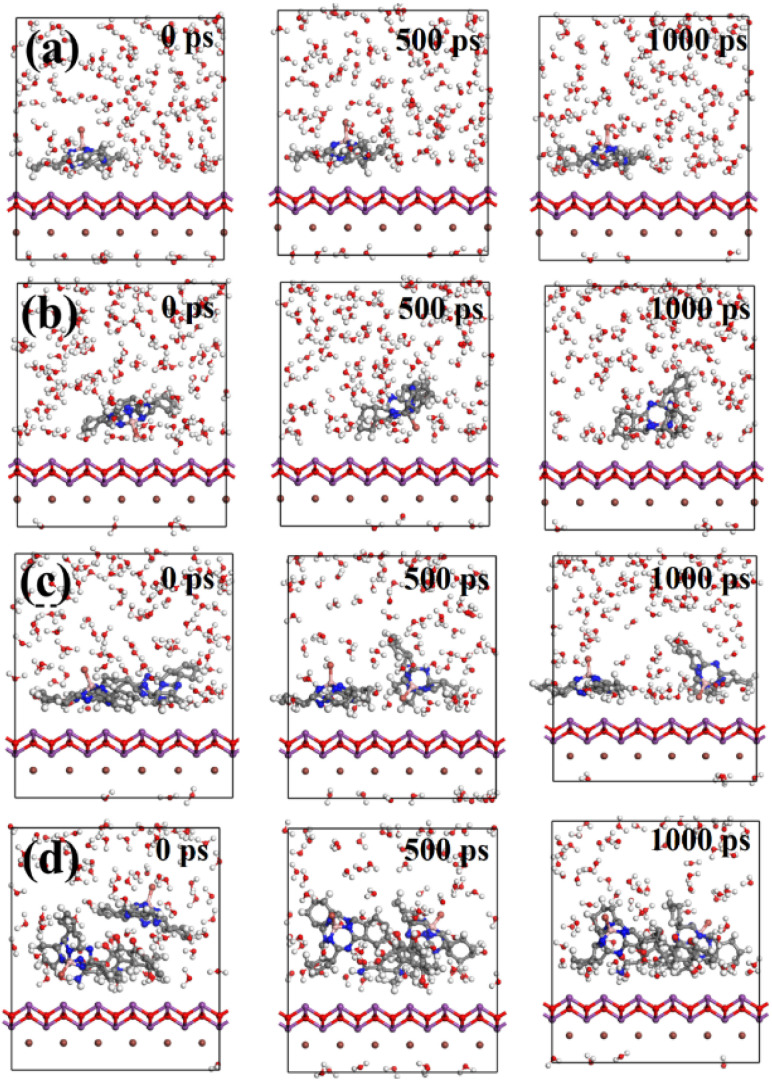
Mainly studied (a and b) Hat-shaped and umbrella-shaped SubPc-Br, (c) SubPc-Br dimer array, (d) the presence of SubPc-Br dimer array/TC on the BiOI (001) surface.

The coupling of SubPc-Br with the BiOI (001) in an aqueous medium was examined using molecular dynamics simulations. Given that SubPc-Br typically exists as a dimer or in molecular arrays, the anchoring mechanism of SubPc-Br dimers on the BiOI (001) was studied. Initially, the trajectory of a lone SubPc-Br unit on the BiOI (001) was examined, as illustrated in Fig. 5a and b. The data demonstrate that SubPc-Br molecules, in both forms, remained stably bound to the BiOI (001) surface across the entire area process (0–1000 ps). This stability is likely due to the van der Waals interactions between the BiOI (001) surface and its benzene rings in SubPc-Br molecules.^66^ Subsequently, dynamics of SubPc-Br dimers and the co-adsorption of SubPc-Br dimers with TC molecules on the BiOI (001) surface were further simulated, as illustrated in Fig. 5b and c. The SubPc-Br dimers were also observed to be stable on the BiOI (001) surface. Notably, the SubPc-Br dimers and TC molecules were found to approach each other over time. This proximity is associated with the π–π stacking intermolecular interactions between TC and SubPc-Br, ensuring that the entire system remained tightly adsorbed on the BiOI surface throughout the process. This adsorptive interaction is facilitated by the van der Waals forces.^67^ Adhering to the same methodology, the motion trajectories of all possible configurations of SubPc-Br and TC on the BiOI (001) were analyzed (Fig. S3, ESI†). The results demonstrated that all individual TC molecules, as well as all conceivable forms of SubPc-Br and TC molecular systems, stably adsorbed onto the BiOI (001) surface. Despite undergoing a series of displacements, such as moving and flipping, during the simulation calculations, the SubPc-Br molecules and TC molecules remained consistently attached to the BiOI (001) surface.

**Fig. 6:**
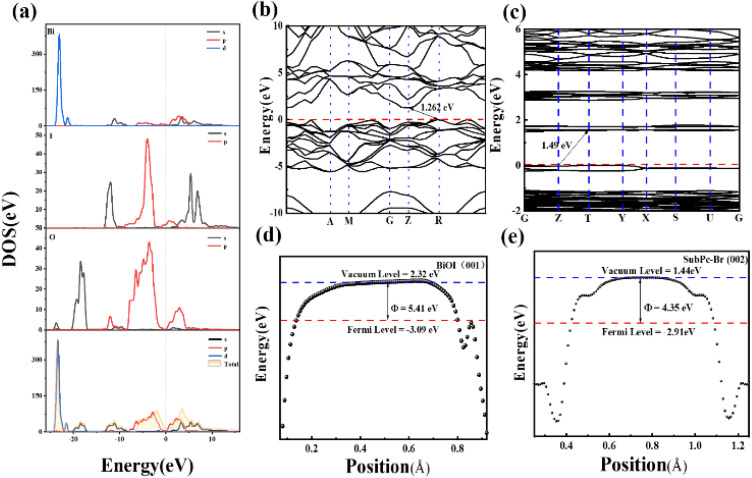
PDOS and TDOS of (a) BiOI, (b) BiOI band structure, the Fermi level is aligned at 0 eV, (c) the energy band diagram of SubPc-Br, (d) the function of BiOI, (e) the function of SubPc-Br.

The total density of states (TDOS) and partial density of states (PDOS) of BiOI were examined to elucidate the contributions to the conduction band minimum (CBM) and valence band maximum (VBM) of the BiOI crystal. The results indicate that the Bi 4f orbitals primarily contribute to these band edges (Fig. 6a).^68^ In addition, Fig. 6b and c illustrate the band structures of BiOI and SubPc-Br, revealing that BiOI is characterized by an indirect bandgap with the VBM located at the *R* point and the CBM at the *Z* point.^69^ In indirect semiconductors such as BiOI, excited electrons must traverse a spatial distance before transitioning to the conduction band. This spatial separation curtails the recombination of photogenerated carriers, thereby improving charge separation efficiency and enhancing photocatalytic performance.^70^ The band diagram of SubPc-Br, as shown in Fig. 6c, reveals a bandgap width of 1.49 eV, with the VBM located at the *T* point and the CBM at the *Z* point. Additionally, Fig. 6d and e depict the work functions and Fermi states of BiOI and SubPc-Br. The Fermi states of BiOI is determined using the following formula:

2 *E*_f_ = *E*_v_ − *W*_F_

The calculation results (Fig. 6c) indicate that BiOI possesses a high work function (5.41 eV) and a low Fermi energy level (*E*_f_) (−3.09 eV). In contrast, SubPc-Br exhibits opposite characteristics, with a work function (*W*_F_) of 4.35 eV and an *E*_f_ of 2.91 eV.

**Fig. 7:**
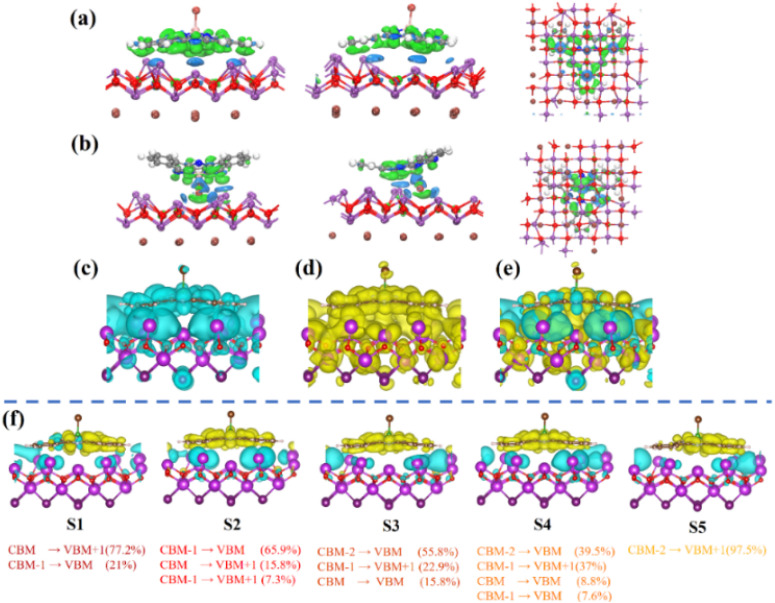
The differential density plot along the *Z*-axis is presented for both the (a) axial up and (b) axial down orientations at three distinct viewing angles. Electron (c) and hole (d) isosurface variation maps of samples, (e) the charge density difference (CDD) of electron transition in samples (isosurface level = 0.005 a.u.). The distribution of electrons (depicted in blue) and holes (depicted in yellow) within the first through fifth excited states of the adsorption structure of (f) is presented.

The 3D charge distribution analysis uncovers the charge arrangement at the interface between SubPc-Br and the BiOI (001) surface, as depicted in Fig. 7a and b (isosurface level = 0.0001 a.u.). In the charge distribution, blue regions indicate charge accumulation, while green regions signify charge depletion. The change in charge density along the *z*-axis at the SubPc-Br/BiOI (001) interface demonstrates that charge redistribution occurs between SubPc-Br and BiOI. The results indicate that a charge difference arises between SubPc-Br and BiOI molecules when electron transfer occurs. This difference significantly suppresses the recombination processes of photoinduced charge carriers. This suggests that electrons are conveyed from SubPc-Br molecules to the BiOI (001) surface, leading to net charge accumulation and the creation of a polarized electrostatic field that facilitates charge separation. The orientation of the internal electrostatic field extends from SubPc-Br to the BiOI surface region, and this field effectively inhibits the recombination of photoexcited carriers. In comparison with BiOI alone, SubPc-Br/BiOI nanocomposites exhibit significantly enhanced carrier mobility, thereby extending carrier lifetime. TDDFT simulations were conducted to explore the electron transfer pathways in the composite material under excitation, thereby further improving its photoelectric properties. A significant absorption peak was observed at 1.22 eV in the simulated optical absorption spectrum of the composite, indicating a shift from the ground state to the S37 excited state (S0 → S37). Fig. 7d and e detail the electron, hole, and charge density distribution (CDD) in the S37 excited state (Fig. S4, ESI†). Fig. 7c and d demonstrate that electrons are predominantly localized at the base of the large ring structure in SubPc-Br and the Bi atoms in BiOI, while holes are concentrated in the [Bi_2_O_2_] layer of BiOI. The charge density distribution (CDD) in Fig. 7e indicates that in the S37 excited state, electron transitions predominantly occur between the nitrogen atoms of the large ring at the base of SubPc-Br and the [Bi_2_O_2_] layer of BiOI. The charge differential distribution of SubPc-Br/BiOI from the first to fifth light-excited states (S1 to S5) was also examined, as illustrated in Fig. 7f. The charge difference density (CDD) diagrams reveal that, irrespective of the specific excited state, electrons are consistently transferred from the [Bi_2_O_2_] layer to the bottom large ring of SubPc-Br (with blue regions indicating electron enrichment and yellow regions indicating hole enrichment). This electron transfer promotes the charge separation process and thereby activates O_2_ into ·O_2_^−^ within SubPc-Br, enabling its participation in photocatalytic reactions and consequently enhancing photocatalytic efficiency. The weak interaction between BiOI and SubPc-Br was analyzed using the Independent Gradient Model (IGM). It was found that, regardless of the form of SubPc-Br, a weak interaction always exists with the BiOI (001) surface (the green region represents van der Waals forces) (Fig S5, ESI†). We've added the precomputed subphthalocyanine fingerprint map to better show the hydrogen – bond and π–π stacking networks in SubPc – Br's packing grid.^56^ The light – green center there marks π–π stacking between SubPc-Br molecules, which promotes their self-assembly on BiOI's (001) surface (Fig S6, ESI†).

### 3.4. Photocatalytic mechanism

**Fig. 8:**
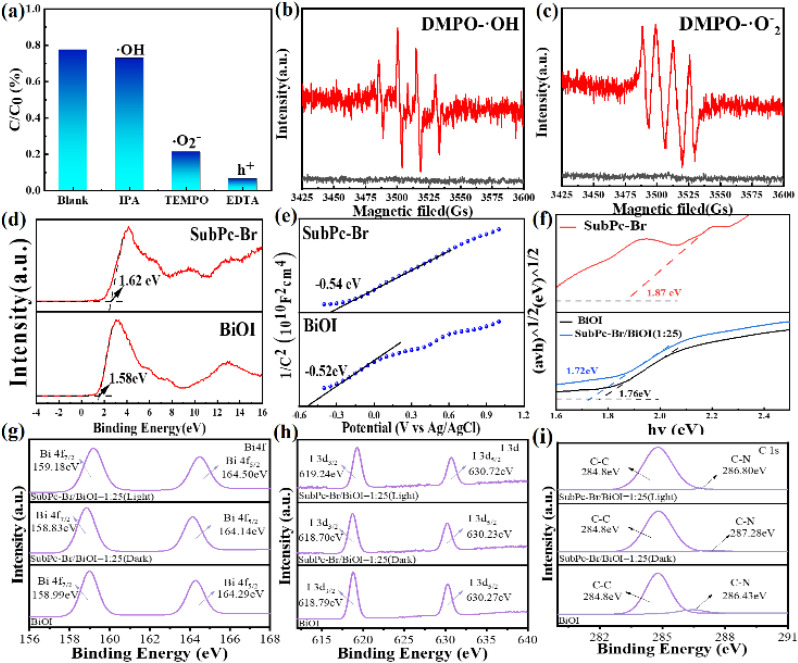
(a) Photocatalytic degradation of TC by SubPc-Br/BiOI and different catcher under equivalent reaction conditions under visible light and dark conditions, (b) EPR spectrum of DMPO-OH and (c) DMPO-O_2_^−^, electronic structure analysis of valence bands using XPS for (d) BiOI and SubPc-Br, (e) Mott–Schottky diagrams of BiOI and SubPc-Br; (f) Tauc's diagrams of BiOI, SubPc-Br and SubPc-Br/BiOI (1 : 25), (g–i) synchrotron radiation XPS features of Bi 4f, I 3d and C 1s of BiOI, SubPc-Br and SubPc-Br/BiOI (1 : 25).

The active oxygen species that primarily participated in the photocatalytic reactions were identified through free radical scavenging experiments. Specifically, hydroxyl radicals (·OH) were scavenged by isopropanol, photogenerated holes (h^+^) were scavenged by ethylenediaminetetraacetic acid (EDTA), and superoxide radicals (·O_2_^−^) were scavenged by 2,2,6,6-tetramethylpiperidinoxyl (TEMPO).^71^Fig. 8a illustrates the degradation of tetracycline (TC) by SubPc-Br/BiOI (1 : 25) in the presence of various scavengers. The results are consistent with the notion that the addition of isopropanol had a negligible effect on the degradation efficiency of tetracycline (TC). However, the introduction of EDTA-2Na and TEMPO induced a marked decrease in degradation efficiency, demonstrating the order of reactivity of the radicals as: h^+^ > ·O_2_^−^ > ·OH. These results indicate that ·O_2_^−^ and h^+^ are the primary reactive species in the SubPc-Br/BiOI (1 : 25) photocatalytic removal of tetracycline through degradation.

Fig. 8b and c display the EPR spectra of DMPO-·OH and DMPO-·O_2_^−^ radicals. In the dark, EPR signal peaks are virtually undetectable. Under illumination, distinctive signals for DMPO-·OH and DMPO-·O_2_^−^ emerge over time, with the signal intensity increasing, and the DMPO-·OH signal peaks persisting for a longer duration. This indicates that superoxide radicals (·O_2_^−^) play a significant participation in the course of the reaction. In general, the valence band potential of the catalyst must be more positive than −0.33 eV to reduce O_2_ to ·O_2_^−^ (O_2_/·O_2_^−^ = −0.33 eV *vs.* NHE). In conjunction with Fig. 8d through Fig. 8f, the VB potentials of BiOI and SubPc-Br are determined to be 1.58 eV and 1.62 eV, in sequence. The flat-band potentials (*E*_fb_) of BiOI and SubPc-Br, as determined by the Mott–Schottky plots, are 0.52 eV and 0.54 eV *vs.* SCE, respectively, which correspond to −0.28 eV and −0.30 eV *vs.* NHE when converted to the standard hydrogen electrode scale.^72^ Considering that the conduction band potential (*E*_CB_) of an n-type heterojunction typically differs from the flat-band potential (*E*_fb_) by 0.2 eV, the values associated with *E*_CB_ BiOI and SubPc-Br are determined to be −0.48 eV and −0.50 eV, respectively. Integrating these findings with the bandgap values presented in Fig. 8f, we deduce that the VB potentials for BiOI and SubPc-Br are 1.28 eV and 1.37 eV, respectively. In summary, the conduction band potentials of BiOI and SubPc-Br are −0.48 eV and −0.50 eV, in parallel, both of which are more negative than the redox potential of O_2_/·O_2_^−^ (−0.33 eV), thus satisfying the conditions for the generation of ·O_2_^−^ and allowing the holes to oxidize H_2_O to ·OH or directly interact with pollutant molecules.^73^ Electron paramagnetic resonance (EPR) spectroscopy corroborates these findings.

Fig. 8g–i present the XPS spectra of Bi 4f and I 3d for BiOI, as well as the C 1s spectra for BiOI and SubPc-Br/BiOI. The results demonstrate electron transfer and the establishment of an electrostatic field within the photocatalyst *versus* the pristine BiOI, in the SubPc-Br/BiOI heterojunctions, the Bi 4f and I 3d states are shifted to lower energy positions, whereas the C 1s state is elevated to a higher energy position. The findings demonstrate that electrons are shuttled from SubPc-Br to BiOI, leading to a decrease in electron density on SubPc-Br and an increase on the BiOI surface. This process establishes an IEF between the two components.^58^ Meanwhile, based on the Fermi level analysis (Fig. 6d and e), BiOI functions as an oxidation photocatalyst (OP) in the system, while SubPc-Br acts as a reduction photocatalyst (RP). Notably, under visible light illumination, the peaks for Bi 4f and I 3d exhibit a redshift, whereas the C 1s peak shows a blueshift. These observations further demonstrate the transfer of photoelectrons under visible light irradiation, electrons are transferred from the oxidation potential (OP) of BiOI to the reduction potential (RP) of SubPc-Br.^74^

**Fig. 9:**
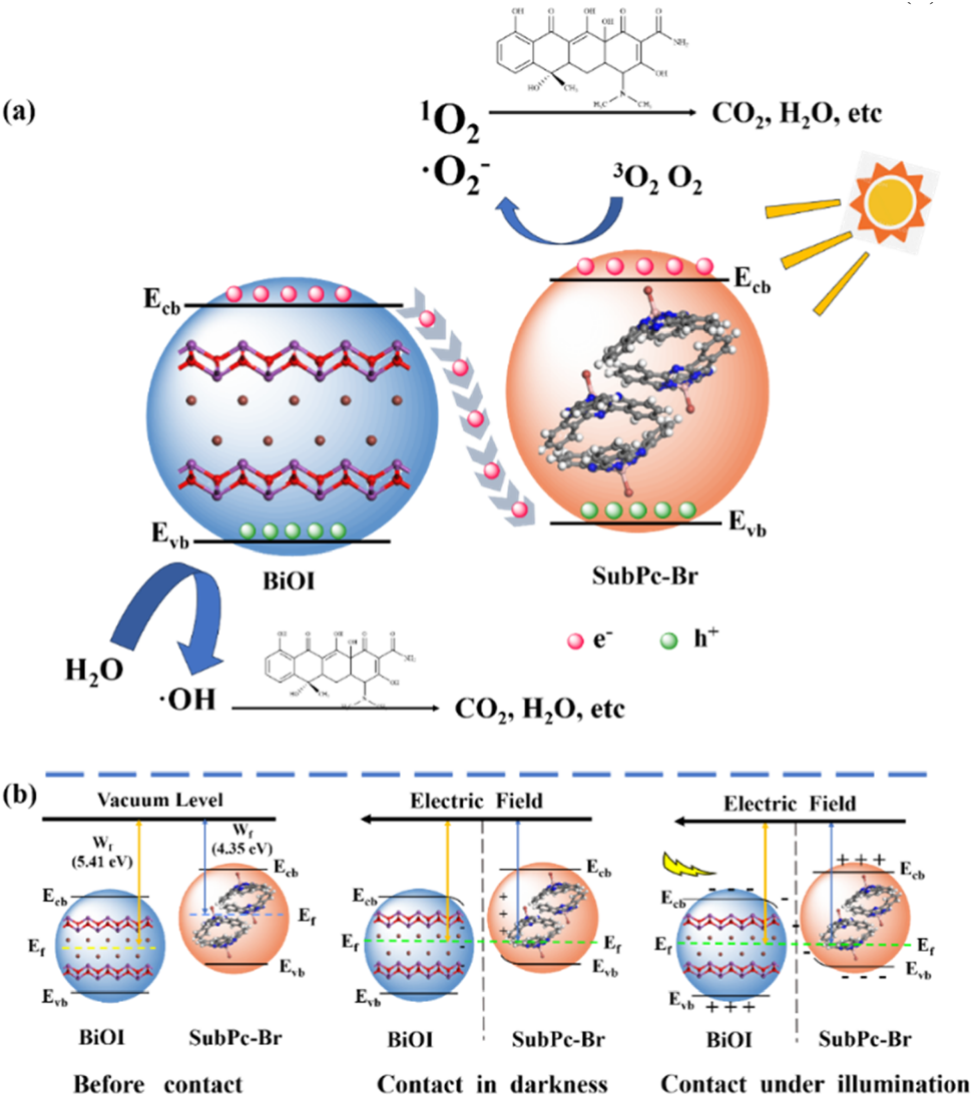
(a) Catalytic oxidation mechanism of the SubPc-Br/BiOI photocatalysts (b) schematic representation of charge transfer in S-scheme heterojunction.

Based on the experimental findings, a catalytic oxidation mechanism for the SubPc-Br/BiOI photocatalyst is synthesized. As illustrated in Fig. 9a, initially, owing to the elevated CB of SubPc-Br relative to that of BiOI, excited electrons from SubPc-Br are injected into BiOI, effectively promoting the efficient separation of photogenerated carriers (chemically reactive (1)). Subsequently, photoexcited electrons on the CB of SubPc-Br further activate dissolved oxygen to form superoxide radicals (·O_2_^−^) (chemically reactive (2)), which oxidize tetracycline (TC) in wastewater. Concurrently, oxygen molecules in SubPc-Br gain energy from the charge transfer, transitioning from the ground state triplet oxygen (^3^O_2_) to the singlet oxygen (^1^O_2_). The addition of ^1^O_2_ and ·O_2_^−^ on SubPc-Br/BiOI photocatalysts enhances the quantum yield of the photocatalytic redox reaction process (chemically reactive (3)).^75^ Concurrently, on the surface layer of bismuth oxyiodide, the photogenerated holes possess sufficient energy to catalyze the oxidation of OH^−^ to ·OH (chemically reactive (4)). Thereafter, these reactive hydroxyl radicals, in conjunction with the holes on the bismuth iodoxide surface, actively participate during the reaction, thereby accelerating the breakdown of tetracycline. This mechanism underscores the crucial role of SubPc-Br/BiOI in photocatalytic mineralization of TC, highlighting reactive oxygen species (ROS) and involvement in achieving efficient pollutant removal (chemically reactive (5)). Moreover, a S-scheme charge transfer pathway is identified between BiOI and SubPc-Br. Initially, the higher CB of SubPc-Br facilitates the injection of excited electrons into BiOI, effectively separating electron–hole pairs. In Fig. 9, when BiOI and SubPc-Br interact, electrons spontaneously transfer from the higher Fermi level (SubPc-Br) to the lower Fermi level (BiOI). This results in electron accumulation at the BiOI interface (bending downward) and electron depletion in SubPc-Br (bending upward) until the Fermi levels are equilibrated. This difference not only promotes the generation of the internal electric field but also effectively suppresses the charge recombination dynamics. The presence of a spontaneous electric field facilitates interfacial charge transfer at the SubPc-Br interface and BiOI. At the interface, an electron-depleted space-charge region and an electron-enriched layer are formed, creating favorable conditions for the transfer and charge separation dynamics of photogenerated charges carriers. Additionally, the endogenous electric field enables efficient transfer of photoelectrons from BiOI to SubPc-Br, leading to the rearrangement of the Fermi characteristic energy levels and bending of the electronic bands in both BiOI and SubPc-Br. This band bending promotes the reorganization of electrons within the conductors of BiOI and SubPc-Br.^76^ Eventually, the conduction band (CB) of SubPc-Br can reduce O_2_ to form superoxide radicals (·O_2_^−^), while the valence band (VB) holes of BiOI are preserved to oxidize H_2_O to hydroxyl radicals (·OH) or directly participate in the photocatalytic degradation of organic pollutants. Thus, the S-scheme heterojunction formed by SubPc-Br/BiOI dramatically improves the separation and migration efficiency of photogenerated charge carriers, thereby boosting photocatalytic performance.^77^ This unique charge transfer mechanism endows SubPc-Br/BiOI photocatalysts with superior redox capability, making them highly effective for the photo-oxidation process degradation of organic contaminants and rendering them of significant practical application value.

1 SubPc-Br/BiOI + *hν* → SubPc-Br/BiOI (h_VB_^+^) + SubPc-Br/BiOI (e_CB_^−^)

2 O_2_ + SubPc-Br/BiOI (e_CB_^−^) → ·O_2_^−^

3 SubPc-Br/BiOI + ^3^O_2_ → SubPc-Br/BiOI + ^1^O_2_

4 OH^−^ + BiOI (h_VB_^+^) → ·OH

5 TC + ^1^O_2_ + ·O_2_^−^ + ·OH + h^+^ → CO_2_ + H_2_O + *etc.*

### 3.5. Degradation pathways

**Fig. 10:**
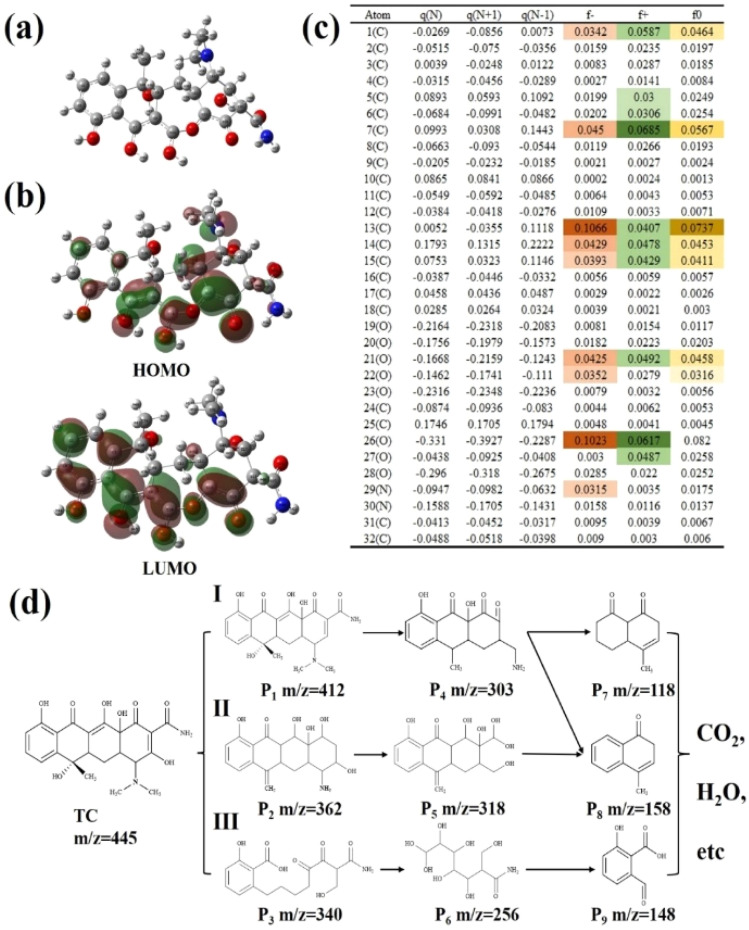
(a) The molecular model of TC, electronic structure of (b) HOMO and LUMO, (c) Hirshfeld charges and localized Fukui functions for TC, with units in elementary charge (*e*), (d) hypothesized photodegradation pathways of TC.

Fig. 10a depicts molecular architecture of tetracycline (TC) with atom numbering. Fig. 10b illustrates the energy states of the HOMO and LUMO for tetracycline (TC) molecules. The DFT calculations were then employed to determine the Fukui function of the TC molecule, as shown in Fig. 10c, providing insights into the electrophilicity at different sites for nucleophilic attack (*f*^−^) and radical attack (*f*^0^). Notably, atoms C1, C7, C13715, O21, O22, O26, and N29 exhibit higher *f*-values, indicating their heightened susceptibility to electrophilic attacks and propensity for electron loss. These atoms also constitute the main components of the HOMO energy level. Additionally, the LUMO region is predominantly located in the areas of C1, C5–7, C13–15, O21, C25, and O27, with their high *f*^+^ values suggesting vulnerability to nucleophilic attacks and ease of electron acquisition. It was also found that C1, C7, C13–C15, and O21–O22, due to their high *f*^0^ values, are more vulnerable to hydroxyl radical attack.

The degradation products of tetracycline (TC) were analyzed at 0-, 15-, 30-, and 45 minutes using LC-MS. By examining the retention times and charge ratios, the mass-to-charge ratios (*m*/*z*) of the intermediates were identified as 412, 303, 118, 362, 318, 158, 340, 256, and 148 (Fig. S7, ESI†). Based on the specific Fukui function analysis and LC-MS detection results, three degradation pathways for TC were put forward. As shown in Fig. 10d, in Pathway I, the intermediate P1 primarily results from dehydrogenation at O23, followed by hydroxylation and cyclolysis to yield low molecular weight ketones or carboxylic acid compounds with *m*/*z* ratios of 303, 158, and 118. Pathway II involves the continuous oxidation of tetracycline (TC) molecules to generate intermediate P2, which is subsequently oxidized by superoxide radicals (·O_2_^−^) and hydroxyl radicals (·OH). This process leads to the formation of various functional groups, primarily through the removal of methyl groups, amino groups (–NH_2_), and hydroxyl groups (–OH), ultimately yielding intermediate P5. Intermediate P5 is further degraded into low molecular weight ketone P8 through a series of dehydroxylation and cyclolysis reactions. Pathway III begins with partial detachment of hydroxyl and amino groups and an opening-ring reaction to form intermediate P3, which is then oxidized to intermediate P6, and finally, P6 undergoes detachment and amine reaction to yield P9. Ultimately, these organic intermediates are further oxidized into carboxylic acid compounds. As the reaction progresses, the resulting intermediates are further oxidized by ·O_2_^−^ and ·OH. Under the continuous action of these radicals, the intermediates undergo further decomposition and are ultimately fully converted to carbon dioxide (CO_2_) and water (H_2_O), along with other small inorganic molecules, achieving complete mineralization.

## 4. Conclusions

In summary, flaky BiOI and SubPc-Br were successfully synthesized using established methods, and the two components were effectively combined through rotary evaporation. It was found that SubPc-Br/BiOI (1 : 25) exhibited a significant catalytic degradation effect on antibiotics in water under sunlight. Within 1 hour, SubPc-Br/BiOI (1 : 25) achieved an 84.4% removal rate of TC in water, which is 1.6 times more efficient than BiOI alone. Moreover, after five cycles, the performance was 2.6 times that of BiOI under the same conditions. The addition of SubPc-Br to the surface enhanced both the catalytic effect and stability of the entire system. The enhanced photocatalytic activity is attributed to the formation of an S-scheme heterojunction between SubPc-Br and BiOI, which establishes an internal electric field, improving electron transfer across the interface while enhancing charge carrier separation. The alteration in the oxidation–reduction potential of the catalyst enables the material to generate more active free radicals capable of degrading tetracycline in practical catalytic applications, thereby improving the photocatalytic effect. By introducing supramolecular SubPc-Br arrays on BiOI nanoparticles, we can enhance the photocatalytic efficiency of materials through the construction of S-scheme heterojunctions, providing a strategy for the development of efficient composites for renewable energy and environmental applications.

## Data availability

Data will be made available on request.

## Author contributions

Yijian Zhou: writing – original draft, investigation, software, data curation. Mengting Ji: investigation. Shengqian Liang: investigation. Jiahang Song: methodology. Haotian Wu: software. Enzhou Liu: supervision, resources. Bing Wang: writing – review & editing, software. Chen Wang: resources, validation. Bo Zhou: software, methodology. Zhuo Li: writing – review & editing, supervision, funding acquisition, project administration, methodology.

## Conflicts of interest

The authors declare that they have no known competing financial interests or personal relationships that could have appeared to influence the work reported in this paper.

## Supplementary Material

RA-015-D5RA02536B-s001
