# Supplementary material for: SubPc-Br/BiOI S-scheme heterojunctions: efficient charge separation for enhanced photocatalytic degradation of tetracycline
Source: RSC Adv. 2025 Jun 2;15(21):16799–813. doi: 10.1039/d5ra02536b (PMC12127920; doi:10.1039/d5ra02536b)
Supplement: RA-015-D5RA02536B-s001 [file RA-015-D5RA02536B-s001.pdf]

Supplementary Information (SI) for RSC Advances.
This journal is © The Royal Society of Chemistry 2025

# Supporting Information

# SubPc-Br/BiOI S-Scheme Heterojunctions: Efficient Charge Separation for Enhanced Photocatalytic Degradation of Tetracycline

Yijian Zhou [a, b, 1], Mengting Ji [a, b, 1], Shengqian Liang [a, b], Jiahang Song [a, b], Haotian Wu [a, b], Enzhou Liu [a], Bing Wang[a, c, *], Chen Wang [a, b], Bo Zhou[d], Zhuo Li [a, b, *]

[a] *School of Chemical Engineering, Northwest University, Xi'an 710069, China*

[b] *International Scientific and Technological Cooperation Base for Clean Utilization of Hydrocarbon Resources, Shaanxi Key Laboratory for Carbon Neutral Technology, Chemical Engineering Research Center of the Ministry of Education for Advance Use Technology of Shanbei Energy, Shaanxi Research Center of Engineering Technology for Clean Coal Conversion, Collaborative Innovation Center for Development of Energy and Chemical Industry in Northern Shaanxi, Xi'an 710069, China*

[c] *Technological Institute of Materials & Energy Science (TIMES), Xijing University, Xi'an 710123, China*

[d] *Institute of Modern Physics, Shaanxi Key Laboratory for Theoretical Physics Frontiers, Northwest University, Xi'an 710069, China*

# S1. Experimental Section

## S1.1. Details of the Characterization Tests

The morphology and microstructure of the photocatalysts were characterized using scanning electron microscopy with energy-dispersive X-ray spectroscopy (SEM-EDS and EDS Mapping, JEOL JSM-6390 system) and transmission electron microscopy (TEM, Talos F200X). The crystallinity of the photocatalysts was assessed using a Rigaku D/max-IIIA X-ray diffractometer (XRD) with Cu Kα radiation, at a scanning rate of 10°/min over a range of 5° to 80°[1]. Fourier-transform infrared spectroscopy (FT-IR) was performed using a Bruker Vector 002 spectrophotometer. X-ray photoelectron spectroscopy (XPS) analysis was conducted on a Kratos AXIS NOVA spectrometer, with calibration against the C 1s peak at 284.8 eV[2].

Ultraviolet-visible (UV-vis) absorption spectra were recorded using a Shimadzu UV-3600 spectrometer, with $BaSO_4$ as the reflectance standard. Photoluminescence (PL) spectra were obtained using a Hitachi F7000 fluorescence spectrophotometer[3]. Time-resolved fluorescence decay spectra were measured using an F-4500 fluorescence spectrophotometer[4]. Electrochemical measurements were performed using a CHI 660E electrochemical workstation in a standard three-electrode system (platinum counter electrode, saturated calomel reference electrode, and sample/FTO working electrode) in a 0.1 M $Na_2SO_4$ aqueous solution, including transient photocurrent response (TPR) and electrochemical impedance spectroscopy (EIS) Nyquist plots. The optical current response was measured at a potential of 0.5 V relative to the saturated calomel electrode (SCE) using a 300 W xenon lamp equipped with a 400 nm filter as the light source[5]. Electron paramagnetic resonance (EPR) spectroscopy, with DMPO as a spin trap, was employed to detect the possible formation of reactive oxygen species ($\cdot OH$ and $\cdot O_2^-$) during the experiments[6].

## S1.2. Details of Theoretical Calculations

The Ewald summation method was employed with a cutoff radius of 9.50 Å, and both bonding and non-bonding effects were set to atom-based summation. The thermostat was maintained at 298 K with a time step of 0.1 fs and a total simulation time of 1000 ps. The Cambridge Sequential Total Energy Package (CASTEP) module was used to compute the electronic structure of BiOI (001), SubPc-Br, and SubPc-Br/BiOI in Materials Studio 2017. This included band structure, density of states, Fermi energy level, and differential charge density. The generalized gradient approximation (GGA) and Perdew-Burke-Ernzerhof (PBE) functional were employed for all structural optimizations and static self-consistent and non-self-consistent calculations. For dispersion correction, Grimme's density functional theory (DFT-D) method was utilized. The plane-wave energy cutoff was set to 500 eV, and the Brillouin zone was sampled using the Monkhorst-Pack scheme with a $1 \times 1 \times 1$ k-point grid. The convergence criteria were set as follows: total energy tolerance of $2.0 \times 10^{-6}$ eV/atom, maximum force tolerance of 0.05 eV/Å, maximum stress

component of 0.1 GPa, and maximum displacement tolerance of 0.002 Å. Prior to molecular dynamics (MD) simulations, the system geometry was optimized at 298 K using the Forcite program and the Nose thermostat to improve accuracy. The MD simulations were conducted using the universal force field, NVT ensemble integration, a time step of 0.1 fs, and a total simulation time of 1000 ps. The adsorption behavior of SubPc-Br on the BiOI (001) surface, the local influence of solvent molecules on the SubPc-Br/BiOI system, and the motion path of the catalyst in the solvent were simulated. The degradation pathway and products of tetracycline (TC) were deduced based on theoretical calculations of HPLC-MS and the Fukui function. Excited states were investigated using time-dependent density functional theory (TD-DFT) with the CAM-B3LYP functional and 6-31G* basis set. The Gaussian software was used to optimize TC and calculate its highest occupied molecular orbital (HOMO) and lowest unoccupied molecular orbital (LUMO) based on DFT, B3LYP functional, and 6-31G* basis set. All CP2K program input files were generated using the Multiwfn program. Additionally, VESTA was combined with Multiwfn for other visual representations.

# S.2 Results

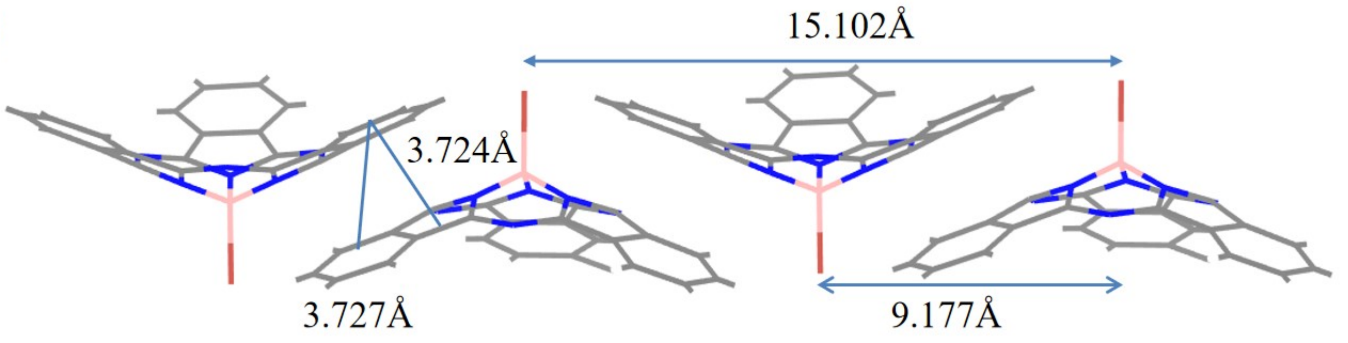


**Fig. S1** Distance analysis between SubPc-Br molecules

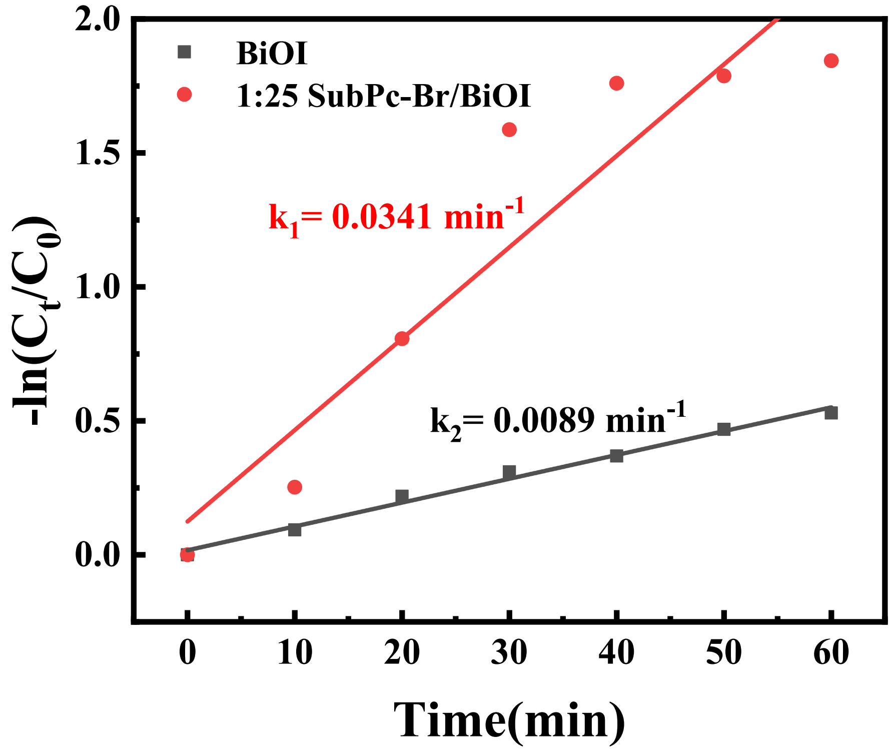


**Fig. S2** BiOI and SubPc-Br/BiOI (1:25) apparent rate constant fitting curve

**Table. S1** Photocatalytic data, mean, and standard deviation

| | Reaction time | First experiment | Second experiment | Third experiment | Average | SD |
|---|---|---|---|---|---|---|
| BiOI | Initial（$C/C_0$） | 1 | 1 | 1 | 1 | 0 |
| | The 30-minute dark reaction was completed.（$C/C_0$） | 0.91121 | 0.94815 | 0.92857 | 0.92931 | 0.01848 |
| | 10-minute light reaction.（$C/C_0$） | 0.80374 | 0.81444 | 0.83013 | 0.8161 | 0.01327 |
| | 20-minute light reaction.（$C/C_0$） | 0.73364 | 0.73889 | 0.72727 | 0.73327 | 0.00582 |

| | | | | | | |
|---|---|---|---|---|---|---|
| | 30-minute light reaction.（$C/C_0$） | 0.69159 | 0.7037 | 0.65909 | 0.68479 | 0.02307 |
| | 40-minute light reaction.（$C/C_0$） | 0.62617 | 0.63185 | 0.61818 | 0.6254 | 0.00687 |
| | 50-minute light reaction.（$C/C_0$） | 0.58879 | 0.57037 | 0.56597 | 0.57504 | 0.0121 |
| | 60-minute light reaction.（$C/C_0$） | 0.54673 | 0.52963 | 0.51299 | 0.52978 | 0.01687 |
| 1:10SubPc-Br/BiOI | Initial（$C/C_0$） | 1 | 1 | 1 | 1 | 0 |
| | The 30-minute dark reaction was completed.（$C/C_0$） | 0.85612 | 0.85069 | 0.86925 | 0.85869 | 0.00954 |
| | 10-minute light reaction.（$C/C_0$） | 0.67626 | 0.67361 | 0.71269 | 0.68752 | 0.02184 |
| | 20-minute light reaction.（$C/C_0$） | 0.56115 | 0.58333 | 0.57313 | 0.57254 | 0.0111 |
| | 30-minute light reaction.（$C/C_0$） | 0.44604 | 0.46181 | 0.42239 | 0.44341 | 0.01984 |
| | 40-minute light reaction.（$C/C_0$） | 0.35971 | 0.36458 | 0.37761 | 0.3673 | 0.00925 |
| | 50-minute light reaction.（$C/C_0$） | 0.32374 | 0.32639 | 0.29552 | 0.31522 | 0.01711 |
| | 60-minute light reaction.（$C/C_0$） | 0.30216 | 0.3125 | 0.32463 | 0.3131 | 0.01125 |
| 1:25SubPc-Br/BiOI | Initial（$C/C_0$） | 1 | 1 | 1 | 1 | 0 |
| | The 30-minute dark reaction was completed.（$C/C_0$） | 0.85186 | 0.87162 | 0.87342 | 0.87324 | 0.01986 |
| | 10-minute light reaction. | 0.47458 | 0.81081 | 0.7943 | 0.46258 | 0.01447 |

| | | | | | | |
|---|---|---|---|---|---|---|
| | (C/$C_0$) | | | | | |
| | 20-minute light reaction. (C/$C_0$) | 0.2178 | 0.73986 | 0.73734 | 0.21785 | 0.01323 |
| | 30-minute light reaction. (C/$C_0$) | 0.17119 | 0.70608 | 0.71519 | 0.18257 | 0.01895 |
| | 40-minute light reaction. (C/$C_0$) | 0.15034 | 0.63892 | 0.61709 | 0.1637 | 0.01194 |
| | 50-minute light reaction. (C/$C_0$) | 0.14492 | 0.61865 | 0.60759 | 0.15287 | 0.00701 |
| | 60-minute light reaction. (C/$C_0$) | 0.12797 | 0.60162 | 0.59177 | 0.13974 | 0.01287 |
| 1:50SubPc-Br/BiOI | Initial (C/$C_0$) | 1 | 1 | 1 | 1 | 0 |
| | The 30-minute dark reaction was completed. (C/$C_0$) | 0.87162 | 0.87342 | 0.85099 | 0.86534 | 0.01246 |
| | 10-minute light reaction. (C/$C_0$) | 0.81081 | 0.7943 | 0.77944 | 0.79485 | 0.01569 |
| | 20-minute light reaction. (C/$C_0$) | 0.73986 | 0.73734 | 0.69662 | 0.72461 | 0.02427 |
| | 30-minute light reaction. (C/$C_0$) | 0.70608 | 0.71519 | 0.66789 | 0.69639 | 0.0251 |
| | 40-minute light reaction. (C/$C_0$) | 0.63892 | 0.61709 | 0.61268 | 0.62289 | 0.01405 |
| | 50-minute light reaction. (C/$C_0$) | 0.61865 | 0.60759 | 0.59155 | 0.60593 | 0.01363 |
| | 60-minute light reaction. (C/$C_0$) | 0.60162 | 0.59177 | 0.57042 | 0.58794 | 0.01595 |
| 1:75SubPc-Br/BiOI | Initial (C/$C_0$) | 1 | 1 | 1 | 1 | 0 |
| | The 30-minute dark reaction | 0.86447 | 0.88302 | 0.87795 | 0.87515 | 0.00959 |

| | | | | | | |
|---|---|---|---|---|---|---|
| | was completed. (C/$C_0$) | | | | | |
| | 10-minute light reaction. (C/$C_0$) | 0.74469 | 0.73585 | 0.77953 | 0.75336 | 0.02309 |
| | 20-minute light reaction. (C/$C_0$) | 0.65282 | 0.66038 | 0.68472 | 0.66597 | 0.01667 |
| | 30-minute light reaction. (C/$C_0$) | 0.52857 | 0.55094 | 0.51969 | 0.53307 | 0.01611 |
| | 40-minute light reaction. (C/$C_0$) | 0.36996 | 0.37736 | 0.38976 | 0.37903 | 0.01001 |
| | 50-minute light reaction. (C/$C_0$) | 0.31502 | 0.31698 | 0.34646 | 0.32615 | 0.01761 |
| | 60-minute light reaction. (C/$C_0$) | 0.26007 | 0.27547 | 0.27559 | 0.27038 | 0.00892 |

**Table. S2** Summary of impactful studies on preparing BiOI-based heterojunctions with different material modifications

| BiOI-based heterojunction | Target degradation compound | degradation time | degradation efficiency | Ref. |
|---|---|---|---|---|
| BiOI/MIL-121 | TC | 120 min | 68% | 7 |
| BiOI-S/BiOI-F | TC | 60 min | 69.43% | 8 |
| 1:10 Cu-Fe/LDH@$BiOI_{1.5}$ | TC | 75 min | 73% | 9 |
| $BiOI/Bi_2O_3$ | TC | 120 min | 80.01% | 10 |
| p-n $BiOI/Bi_3O_4Cl$ | TC | 180 min | 73.5% | 11 |
| $BiOI/Bi_2O_2[BO_2(OH)]$ | TC | 120 min | 70.69% | 12 |
| 1:25 SubPc-Br/BiOI | TC | 60 min | 84.4% | This work |

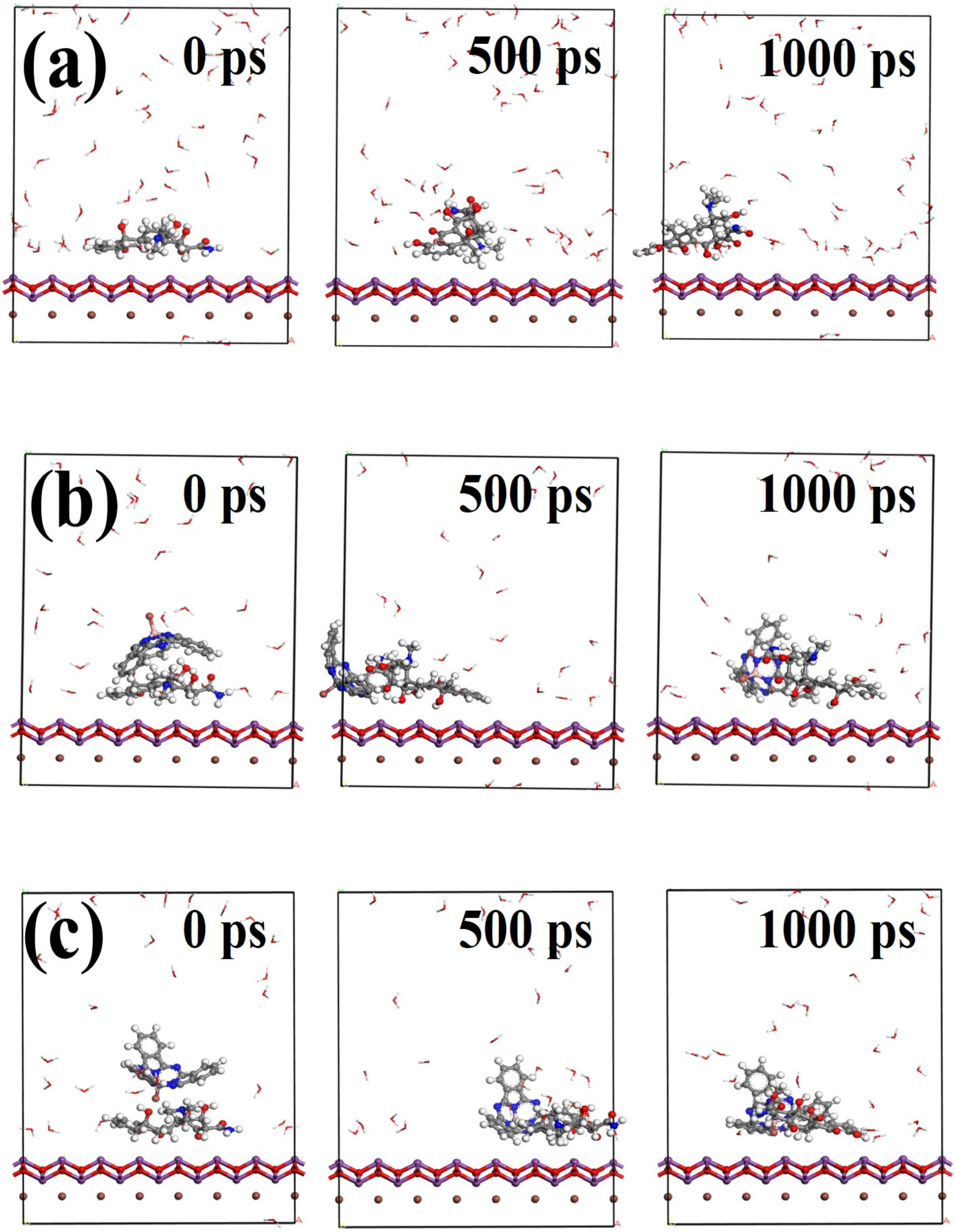


**Fig. S3** Motion trajectories of (a) TC molecules, (b) “axially upward” SubPc-Br molecules and TC molecules, (c) “axially downward” SubPc-Br molecules and TC molecules in the BiOI(001) surface

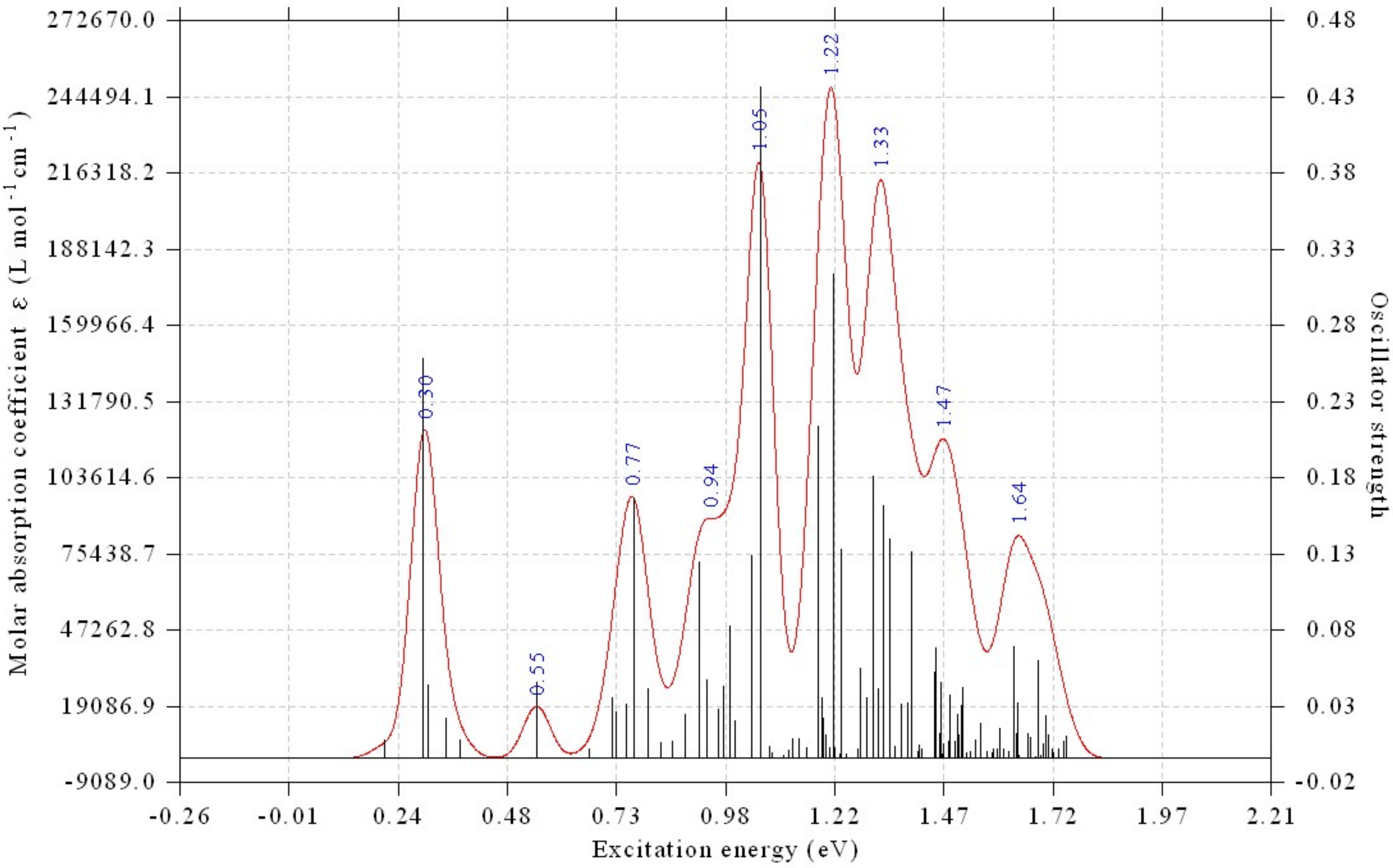


**Fig. S4** Electronic spectroscopy of SubPc-Br/BiOI (1:25)

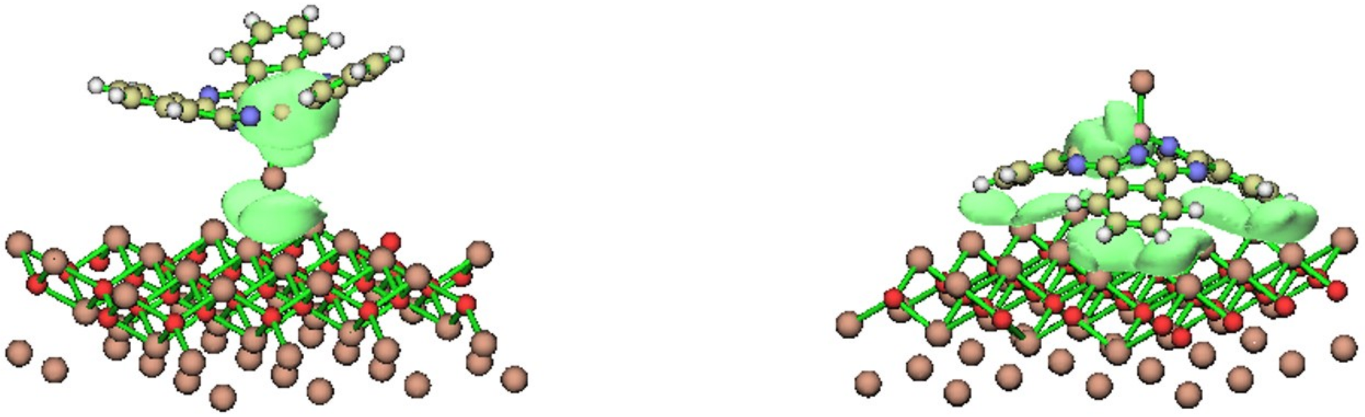

**Fig. S5** The IGM analysis of SubPc-Br and BiOI

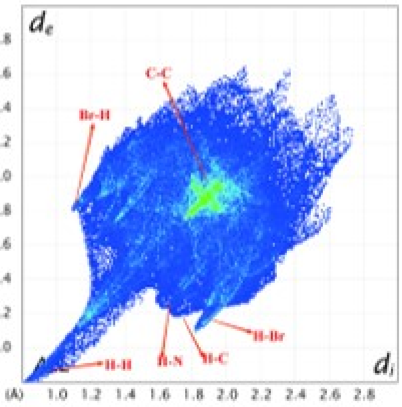


**Fig.S6 two-dimensional fingerprint plot of SubPc-Br [4]**

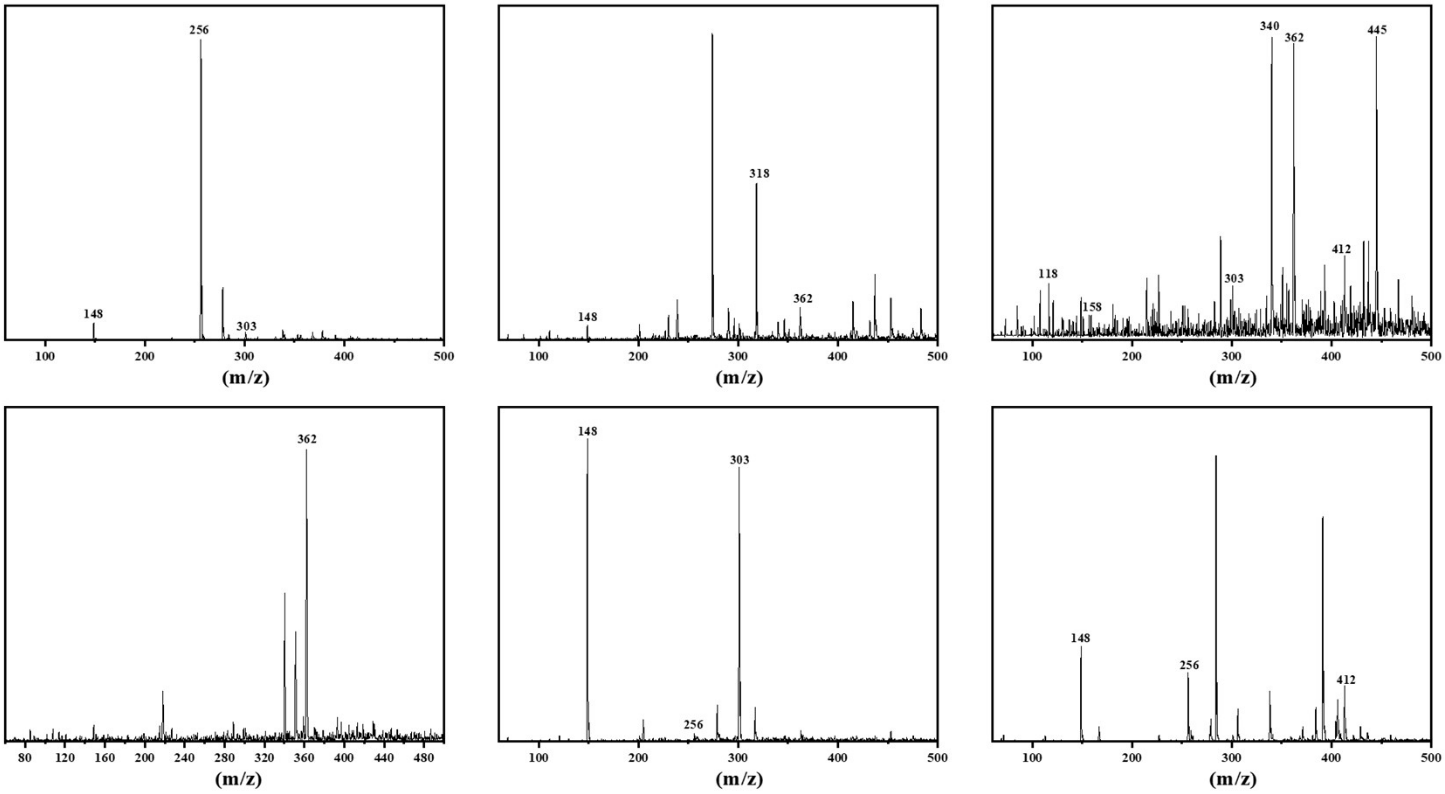


**Fig. S7** HPLC-MS spectra of the TC degradation intermediates.

# References


1. B. Wang, X. Zhang, R. Zhang, Z. Li, B. Tian, H. Ma, Z. Zheng, B. Zhou, M. Ji, C.

Shi and H. Hao, *Chemical Engineering Journal*, 2023, **477**.
2. B. Wang, R. Zhang, Z. Li, C. Shi, E. Liu, Z. Zheng, B. Zhou, M. Ji and H. Chen, *Applied Surface Science*, 2023, **641**.
3. Z. Zheng, B. Wang, Z. Li, H. Hao, C. Wei, W. Luo, L. Jiao, S. Zhang, B. Zhou and X. Ma, *Small*, 2024, **20**, e2306820.
4. M. Ji, B. Wang, Z. Zheng, E. Liu, C. Shi, C. Wang, B. Tian, H. Ma, C. Wei, B. Zhou and Z. Li, *J Colloid Interface Sci*, 2024, **663**, 421-435.
5. B. Wang, Z. Li, H. Ma, J. Zhang, L. Jiao, H. Hao, E. Liu, L. Xu, C. Wang, B. Zhou and X. Ma, *Applied Catalysis B: Environmental*, 2022, **318**.
6. B. Wang, C. Bozal-Ginesta, R. Zhang, B. Zhou, H. Ma, L. Jiao, L. Xu, E. Liu, C. Wang and Z. Li, *Applied Catalysis B: Environmental*, 2021, **298**.
7. D. Dai, J. Qiu, M. Li, J. Xu, L. Zhang and J. Yao, *Journal of Alloys and Compounds*, 2021, **872**.
8. M. Qiao, H. Liu, J. Lv, G. Xu, X. Zhang, X. Shu and Y. Wu, *Nano*, 2019, **14**.
9. Z. Zhu, R. Yang, C. Zhu, C. Hu and B. Liu, *Advanced Powder Technology*, 2021, **32**, 2311-2321.
10. P. Lu, Y. Peng, Y. Yang, S. Chen, J. Shang, C. Yang, M. Xu, J. Bai, Z. Zhao and X. Hu, *Journal of Environmental Chemical Engineering*, 2024, **12**.
11. L. Huang, L. Yang, Y. Li, C. Wang, Y. Xu, L. Huang and Y. Song, *Applied Surface Science*, 2020, **527**.
12. W. Yu, N. Ji, N. Tian, L. Bai, H. Ou and H. Huang, *Colloids and Surfaces A: Physicochemical and Engineering Aspects*, 2020, **603**.
